# Impact of Immunosuppressive Drugs on Fibroblasts: An In Vitro Study

**DOI:** 10.3390/jcm11113107

**Published:** 2022-05-31

**Authors:** Gunar Wagner, Lisa Sievers, Malte Tiburcy, Wolfram Hubertus Zimmermann, Otto Kollmar, Gerhard Schmalz, Dirk Ziebolz

**Affiliations:** 1 Department of Restorative Dentistry and Periodontology, University Medical Center Leipzig, 04103 Leipzig, Germany; wagnergu@medizin.uni-leipzig.de; 2 Department of Preventive Dentistry, Periodontology and Cariology, University Medical Centre Goettingen, 37075 Goettingen, Germany; lisa_sievers@gmx.de; 3 Institute of Pharmacology and Toxicology, University Medical Center Goettingen, 37075 Goettingen, Germany; m.tiburcy@med.uni-goettingen.de (M.T.); w.zimmermann@med.uni-goettingen.de (W.H.Z.); 4 Department of General and Visceral Surgery, University Hospital of Basel, 4002 Basel, Switzerland; otto.kollmar@clarunis.ch

**Keywords:** immunosuppressive drugs, drug-induced gingival overgrowth, periodontal disease

## Abstract

Background: The aim of this study was to compare the direct impact of different agents for immunosuppressive therapy on mouse fibroblasts as a possible cause of drug-induced gingival overgrowth (DIGO). Methods: 3T3 mouse fibroblasts were cultivated in cell-specific media (2 × 10^4^ cells/mL) and treated for 6, 24, 48 and 72 h with one of three immunosuppressive drugs (IsDs): cyclosporin a (CsA), tacrolimus (TaC) and sirolimus (SiR). Different concentrations (10–750 ng/mL) were used to mimic serum levels under active immunosuppressive therapy conditions. Cell population characteristics (cell number, viability and morphology) were assessed using computer-assisted cell analysis. Expression of pro-collagen type I carboxy-terminal propeptide (PICP) was identified using an ELISA assay. Results: The influence of IsDs on the biological status of 3T3 fibroblasts was time- and dose-dependent. Comparing CsA and TaC, the total cell amount was enhanced using concentrations in the range of 10–150 ng/mL (*p* > 0.05). In contrast, treatment with SiR resulted in a decrease in the average cell number (*p* < 0.01). PICP and cell diameter of fibroblasts were not susceptible to IsD treatment (*p* > 0.05). Conclusions: Our results revealed time-dependent effects of IsDs, with distinct influences on cell number. The cell morphology and the PICP balance of the investigated fibroblast cell line remained unaffected. Hence, the potential role of IsDs is not a unilateral mechanism of action but rather a multifactorial process.

## 1. Introduction

Autoimmune disease affects as much as 4.5 percent of the world’s population, and more than 81 variants were recently identified [1]. Patients suffering from these diseases or in need of solid organ transplantation (SOT) are frequently administered pharmacological immunosuppressive therapy. Several immunosuppressive drugs (IsDs) with different molecular interactions have evolved to influence the inert immunological defense on the recipient patient to secure safety from various detrimental factors [2]. Post-transplant patients face multiple life-threatening complications and various impairments in their quality of life [3]. Manifestation of these systemic conditions or drug therapies has been found in the oral cavity, leading to dental or periodontal diseases, as well as oral mucosal lesions [4,5]. These effects must be differentiated from gingival diseases that are induced by biofilm formation. The extent, severity and progression of such lesions could be affected by local predisposing, as well as systemic modifying factors [6].

In this context, the aspect of drug-induced gingival overgrowth (DIGO) must be considered, as it represents a major burden on the quality of life of patients, challenging their dental oral health [7]. To highlight the periodontal side effects and oral pathogenesis of IsDs, clinical investigations on calcium-channel blocker were conducted [8]. It was revealed that the prevalence of DIGO in patients taking IsDs varies depending on the immunosuppressive medication [9]. Cyclosporine (CsA) is widely used and was reported to be associated most frequently with DIGO (60–70%) [10]. The mean DIGO index was significantly lower in patients receiving therapy with tacrolimus (TaC) (15–28.9%) [11,12]. Alternative drugs, such as sirolimus (SiR), are less toxic and represent more therapeutic options. Nevertheless, it proportionately leads to gingival overgrowth (15.6%) [13]. The precise mechanisms of drug-induced gingival overgrowth are not fully elucidated. A multifactorial model expanding on the interaction between metabolites and gingival fibroblasts affected by pharmacokinetic variables was presumed [14]. Direct effects of cyclosporine metabolites on gingival fibroblasts, protein synthesis, apoptosis and collagen production have been confirmed [15]. Additionally, it has been speculated that hyperproliferation of fibroblasts with a deposition of extracellular matrix leads to DIGO [16]. To reveal the distinctive influences of three immunosuppressive drugs—cyclosporin A (CsA), tacrolimus (TaC) and sirolimus (SiR)—on fibroblasts, a comparative in vitro study was conducted. The aim of this in vitro study was to analyze the pharmacological impact on cell number, viability and synthesis of collagen using procollagen type I carboxy-terminal propeptide (PICP) production as an outcome parameter. We speculated that cell morphological changes in fibroblast have direct implications for the onset of DIGO under conditions with distinct pharmaceutical concentrations.

## 2. Materials and Methods

### 2.1. Study Design

To reveal the effect of different immunosuppressive drugs on fibroblasts, a comparative in vitro study was conducted. The experimental setup should reveal possible effects of dose differences in a time-dependent manner. For this in vitro study, mouse fibroblast cells (3T3) from passage 39 were used.

### 2.2. Immunosuppressive Drugs

The pharmaceutical preparations used in this in vitro study were commercially avail-able IsDs provided by Merck Millipore KGaA (Darmstadt, Germany) for experimental purposes. Cyclosporin (CsA; Sandimmun^®^), tacrolimus (TaC; Prograf^®^) and sirolimus (SiR; Rapamune^®^) (Merck Millipore, Burlington, MA, USA) were used with dimethyl sulfoxide (DMSO) (Merck Millipore KGaA, Darmstadt, Germany) as a solvent according to the manufacturer’s specifications. The different IsDs were tested in three different concentrations, which were comparable to clinical whole-blood trough levels. For the treatment with CsA, concentrations of 15, 150 and 750 ng/mL were chosen. TaC and SiR were used in concentrations of 10, 15 and 20 ng/mL.

### 2.3. Cell Culture and Treatment

Cell cultivation was performed using standardized methods under sterile conditions for Mouse embryo fibroblasts (3T3) [17]. The fibroblasts were maintained in a humidified atmosphere of 5% CO_2_ at 37 °C and replicated in a DMEM medium combined with 10% fetal bovine serum (FBS), 50 ng/mL amphotericin B (PAN Biotech GmbH, Aidenbach, Germany) and 50 μg/mL penicillin/streptomycin (Gibco by life science, Invitrogen, Carlsberg, Gibco Life Technologies (acquired by Thermo Fisher), Waltham, MA, USA). Cells were subjected to infection serology tests for bacteria, fungi, mycoplasms, HIV-DNA and hepatitis B/C-DNA and classified as negative. Culture medium was replaced by fresh culture medium every two or three days and cultured to 70% confluence. Mouse fibroblast cells (3T3) from passage 39 were seeded into 24-well plates at a concentration of 2 × 10^4^ /mL until adherence was achieved 48 h later and then further processed for experiments. Cell treatment with IsDs was conducted with concentrations comparable to clinical whole-blood trough levels. For treatment with CsA, concentrations of 15, 150 and 750 ng/mL were chosen for the individual group. The two immunosuppressants, TaC and SiR, were used in concentrations of 10, 15 and 20 ng/mL. Inoculation of the cells was performed for 6, 24, 48 or 72 h for each experimental group. Medium was routinely exchanged after 24 h and replaced with the individual concentration within the respective experimental group to ensure proper contact time. A periodic microscopic control was performed at defined observation periods to evaluate morphological changes and cell confluence in order to confirm the cell proliferation rate (Axiovert 200M, Zeiss, Göttingen, Germany). All tests were performed in duplicate and repeated twelve times. For further analysis, cells were rinsed with phosphate-buffered saline (PBS) and suspended in culture medium.

### 2.4. Cellular Analysis

The cell number, viability and mean cell diameter were determined using computer-assisted cell analysis (CASY^®^, Modell TT, Roche Diagnostics GmbH, Penzberg, Germany). All cells from the experiments were suspended in 1 mL of DMEM after centrifugation for cell-counting purposes. A simultaneous determination of the number of living cells in relation to the total cell number (viability) was achieved without trypan blue staining. Cell concentration was generated automatically, and the dilution factor was considered. The generated data (cell number, viability and mean diameter) were transferred using Microsoft Excel^®^. Cell morphology was determined at defined time points using light microscopic imaging (Axiovert 200M, Zeiss, Göttingen, Germany). The control groups (CTRL) consisted of DMSO without the addition of IsDs.

### 2.5. Procollagen Type 1 Assay (PICP)

**Figure 1:**
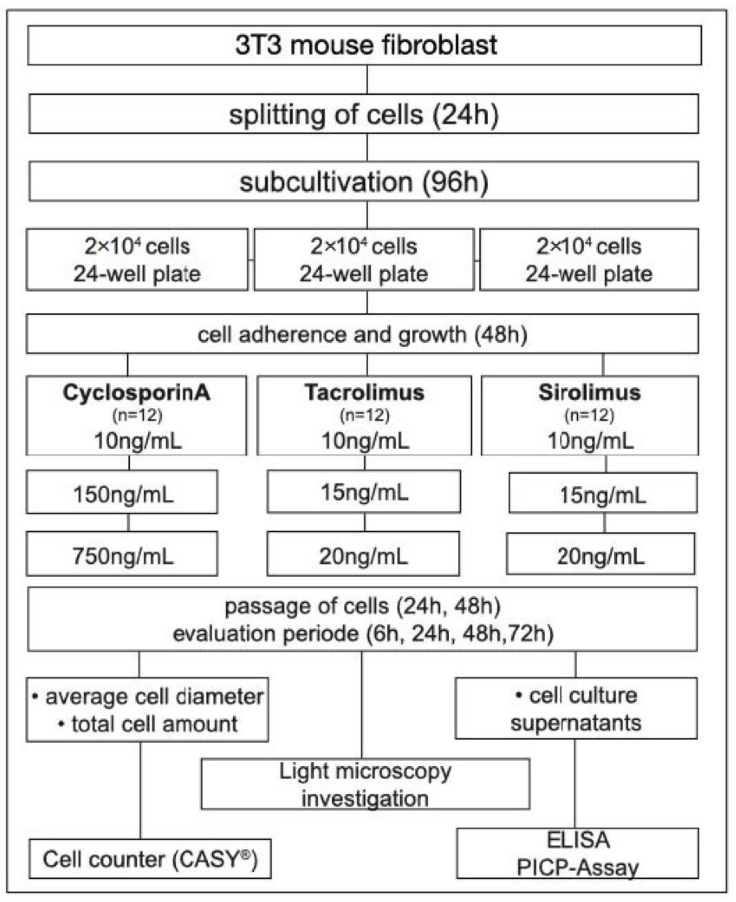
Flow chart of the experimental design.

To evaluate potential shifts under the influence of IsDs in fibroblast collagen synthesis an enzyme-linked immunoabsorbant assay (ELISA) was performed. A competitive ELISA with high sensitivity for mouse procollagen type I carboxy-terminal propeptide (PICP) was used (BlueGene Biotech Co., Shanghai, China). For the analysis of PICP cell culture, supernatants were used within the evaluation periods of 6 and 48 h. Density measurements at 450 nm were conducted with the aid of a spectrophotometer (Spektra Max M2, Molecular Devices, San Jose, CA, USA). Curve fitting was applied using Soft Max Pro 4.8 software, and data were further processed using Microsoft Excel^®^. The experimental settings are displayed in a flow chart in Figure 1.

### 2.6. Statistical Analysis

For statistics, GraphPad Prism9 software (GraphPad Software, San Diego, CA, USA) was used. Statistical comparison was achieved by two-way analysis of variance (ANOVA). No mathematical correction was made for multiple comparison. All graphs are presented as mean ± SEM.

## 3. Results

### 3.1. CsA

**Figure 2:**
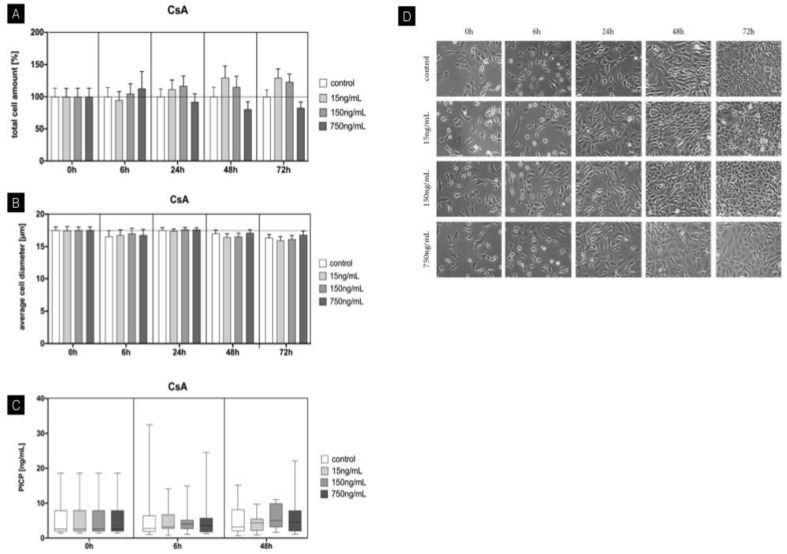
Time-dependent influence of cyclosporin A on mouse fibroblasts. (**A**) Total cell amount (baseline-corrected). Upregulation of cell amount after 24 h using low and intermediate concentrations of 15 ng/mL and 150 ng/Ml. (**B**) Average cell diameter showing no significant influence of CsA. (**C**) Procollagen concentration with no significant changes using different concentrations of CsA. (**D**) Cell morphology evaluation using light microscopy between 6 h and 72 h. Overall increased cell confluence with longer incubation periods. Concentration of CsA with decreased cell confluence at 48 h and 72 h using high concentrations of 750 ng/mL. Columns and error bars represent the mean ± SEM (n = 12); significant (*p* < 0.05).

CsA was accountable for an increase in total cell amount of fibroblasts. Baseline corrected values of low (15 ng/mL) and intermediate (150 ng/mL) concentrations result-ed in an increase of up to 25% after 24 h. This was confirmed using light microscopic analysis, showing an enhanced cell confluence within these groups. High concentrations (750 ng/mL) did not lead to an upregulation but rather a reduction in total cell amount after an inoculation period of 24 h. After 72 h, cell confluence was lowest using CsA at high concentrations, confirming these results. The average cell diameter remained unaffected by time and concentration with CsA treatment. Likewise, no significant influences on PICP synthesis were identified under CsA treatment. (Figure 2).

### 3.2. TaC

**Figure 3:**
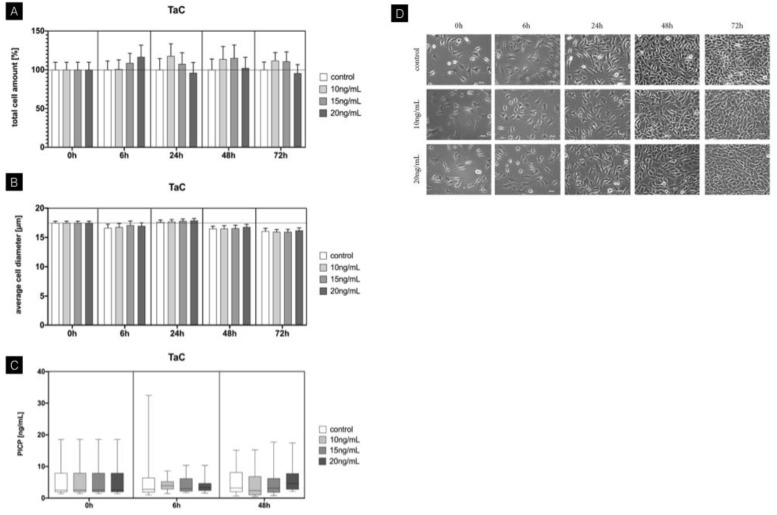
Time-dependent influence of tacrolimus on mouse fibroblasts. (**A**) Total cell amount (baseline-corrected). Upregulation after 6 h at intermediate and high concentrations of 15 ng/mL and 20 ng/mL. Persistent increase in cell amount after 24 h using low and intermediate concentrations. No significant changes using high concentrations of 20 ng/mL after 24 h. (**B**) Average cell diameter was slightly reduced using TaC after 48 h for all concentrations. (**C**) Procollagen concentration with no significant changes using different concentrations of TaC. (**D**) Cell morphology evaluation using light microscopy between 6 h and 72 h. Overall increased cell confluence with longer inoculation periods. Columns and error bars represent the mean ± SEM (n = 12); significant (*p* < 0.05).

Regarding the influence of TaC on total cell amount of fibroblasts, an upregulation of up to 15% was evident compared to the control after 24 h using concentrations of 10 ng/mL and 15 ng/mL. In contrast to CsA, the average cell diameter was slightly reduced after 48 h for all concentrations using TaC. Cell morphology evaluated with light microscopy showed an overall increase in cell confluence with longer inoculation periods with TaC, but no concentration-dependent effects were observed. PICP synthesis was not affected by TaC with different concentrations (Figure 3).

### 3.3. SiR

**Figure 4:**
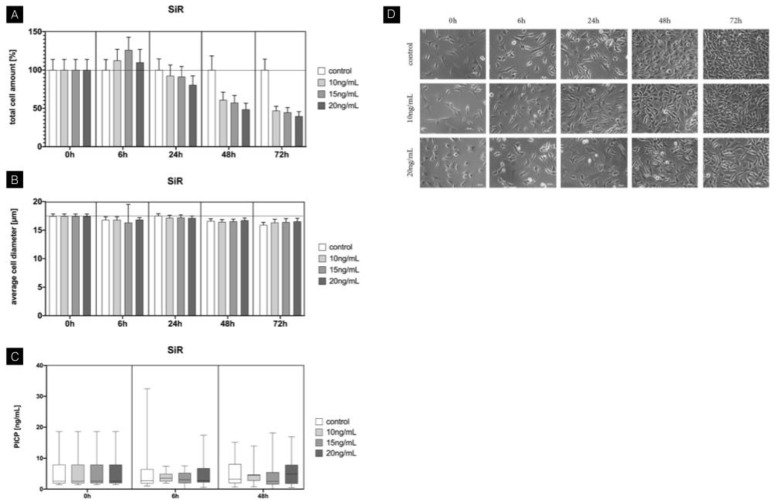
Time-dependent influence of sirolimus on mouse fibroblasts. (**A**) Total cell amount (baseline-corrected). Constant downregulation after 24 h along with increased concentrations. Drop in total cell amount after 72 h independent of concentration. (**B**) Average cell diameter showing no significant influence of SiR. (**C**) Procollagen concentration with no significant changes using different concentrations of SiR. (**D**) Cell morphology evaluation using light microscopy between 6 h and 72 h. Overall increased cell confluence with longer incubation periods. Reduced cell confluence within the same period showing concentration-dependent effects of SiR. Columns and error bars represent the mean ± SEM (n = 12); significant (*p* < 0.05).

The analysis regarding total cell amount revealed a significant influence of SiR on fibroblasts. A reduction in cell number of up to 50% was evident compared to the control after 72 h (*p* < 0.05). Light microscopy revealed an overall increase in cell confluence over time for fibroblasts treated with SiR. Within the groups, after 48 h and 72 h, cell confluence was reduced compared to the control. No significant differences were observed at any time point for low and high concentrations regarding average cell diameter. A PICP assay did not reveal any influence of SiR on fibroblasts with concentrations between 10 and 20 ng/mL at any time during the investigation period (Figure 4).

## 4. Discussion

It is known that local and systemic factors could lead to increased gingival over-growth. One of the associations uncovered is a plaque-induced inflammatory process that can be modified by medications and should be differentiated from non-plaque-induced etiologies [18]. The administration of medications, mainly calcineurin inhibitors, such as CsA, as the standard first-line treatment for graft rejection prevention in transplant patients have been shown to be responsible for drug-induced gingival overgrowth (DIGO). This adverse reaction in patients seems to be dose-dependent. Moreover, a combination of IsDs and calcium channel blockers appear to particularly influence the periodontal microbiological status [6]. Although microbial dysbiosis promotes DIGO, it is speculated that gingival overgrowth is a consequence of a deregulated immune response and host-derived factors altering tissue homeostasis [19]. Within the scope of efforts to determine the dose-dependent effects of CsA on human gingival fibroblasts (HGFs), heterogeneous results have been achieved. Low doses (≤200 ng/mL) were reported to stimulate proliferation, whereas higher doses (400–800 ng/mL) inhibited proliferation [20]. According to these studies, the current investigation could confirm an increase in total cell amount using CsA at low doses (15–150 ng/mL), along with a reduction using high concentrations of up to 750 ng/mL. The pharmacodynamics of CsA suggests long-term effects influencing the severity of DIGO [21]. Data obtained in the current study were able to confirm a time-dependent influence that became apparent after 24 h inoculation using CsA or TaC. On the other hand, long-term exposure to a low dose of CsA showed no effects on viability and proliferation of HGFs [22]. These findings suggest that decreased apoptosis plays a more important role than increased cell proliferation in the CsA-induced overgrowth of human gingival fibroblasts [15]. Direct effects of cyclosporine metabolites on gingival fibroblasts with increased protein expression and collagen production were investigated in another research approach [23]. Current investigations focus on the inductive effects of CsA on molecular biomarkers responsible for cell–cell adhesion, extracellular matrix structural constituents, transmembrane receptors and basement membrane constituents using real-time PCR. Downregulation of extracellular matrix proteins and inhibition of matrix proteases in the gingival connective tissue are directly related to administration of high-dosage CsA [24]. Additionally, modulation of the fibrosis response in gingival fibroblasts were with deposition of fibrotic tissue through the extracellular matrix (ECM) [16]. The current study did not confirm changes in cell morphology or collagen synthesis during CsA treatment under selected conditions. Similarly, other IsDs could possibly have a different impact on these outcome parameters. Increased levels of matrix metalloproteinases (MMPs) were observed with administration of TaC (≤800 ng/mL). No synergistic induction of interstitial collagen overexpression by gingival fibroblasts is expected [25]. These findings are in line with those of the current study, demonstrating no impact of TaC on procollagen synthesis within gingival fibroblasts. In vivo experiments with long-term administration of TaC for up to 240 days revealed deleterious side effects on volume densities and collagen fiber accumulation in the gingival epithelium; these results appeared to be time-dependent [26]. In contrast to CsA and TaC, no associations were revealed for SiR with respect to the severity of DIGO [27]. Nonetheless, SiR seemed to have an impact on cell number of fibroblasts using different dosages (10–20 ng/mL). It must be considered that organ transplantation patients may have increased/decreased dose-adjustment trough concentrations depending on their genotype. Some genes, e.g., IL-10, were shown to have a substantial impact on SiR and TaC dosing [28,29]. Such pharmacogenetic patterns could have decisive effects on gingival fibroblast and the likelihood for the onset of DIGO. We must conclude that the administration of a specific drug with different concentrations could not be the exclusive factor influencing the likelihood of gingival overgrowth. The underlying genetic, immunological and pathological mechanisms must be considered as a multifactorial causal connection.

### Limitations

To the best of our knowledge, this is the first comprehensive investigation of three different IsDs with varying concentrations to reveal possible basic cellular mechanisms on fibroblasts. The variability of results within the scope of DIGO-associated IsDs may result from differences between in vivo and in vitro studies. Type, origin and passage of the cells (in vitro study), as well as intrinsic differences among subjects (in vivo study), could determine the distinct action of cyclosporine and other IsDs. Due to the experimental setting and the cell type, a sample size of n = 12 was used, although this is not common practice. A major limitation of this study is that we focused on 3T3 mouse fibroblasts. To achieve a better understanding of DIGO, cells from oral mucosa or gingival fibroblasts should be investigated. Additionally, several parameters might influence the occurrence of DIGO (e.g., the relationship between cell number and diameter). Therefore, the generalization of the results of the present investigation from bench to bedside remains speculative, as the clinical phenotype may vary. Additionally, multiple factors were not studied. Mutual influences of microorganisms and the immune system could be decisive for the pattern of disease. However, the use of three different IsDs under controlled experimental conditions allows for a direct comparison on the inductive effects of Csa, TaC and SiR on fibroblasts in general to draw some initial conclusions. Recent scientific developments put an emphasis on genomics for drug administration in patients. The implementation of pharmacogenetics in the context of personalized medicine aims to optimize the outcome of drug response and toxicity, as well as to prevent possible adverse effects [30]. Detecting gene polymorphisms could be helpful in predicting patients with a higher risk of gingival overgrowth [31]. Robust evidence led by the clinical pharmacogenetics implementation consortium (CPIC) must be established to provide clinicians with peer-reviewed guidelines and help to translate laboratory test results into actionable prescribing decisions for specific drugs, such as immunosuppressants [32].

## 5. Conclusions

This study confirmed specific influences of IsDs on mouse fibroblasts. Concentration-dependent effects on cell proliferation became evident, whereas cell morphology and PICP expression remained unaffected. Clinical implications derived from this analysis indicate that within the scope of efforts to reveal the onset of DIGO, the focus should be a multifactorial approach to investigate clinical and pharmacogenetic patterns.

## Data Availability

The datasets used and/or analysed during the current study available from the corresponding author on reasonable request.

## References

[1] Hayter S.M., Cook M.C. (2012). Updated assessment of the prevalence, spectrum and case definition of autoimmune disease. Autoimmun. Rev..

[2] Cajanding R. (2018). Immunosuppression following organ transplantation. Part 1: Mechanisms and immunosuppressive agents. Br. J. Nurs..

[3] Cajanding R. (2018). Immunosuppression following organ transplantation. Part 2: Complications and their management. Br. J. Nurs..

[4] Petti S., Polimeni A., Berloco P.B., Scully C. (2013). Orofacial diseases in solid organ and hematopoietic stem cell transplant recipients. Oral Dis..

[5] Schmalz G., Wendorff H., Berisha L., Meisel A., Widmer F., Marcinkowski A., Teschler H., Sommerwerck U., Haak R., Kollmar O. (2018). Association between the time after transplantation and different immunosuppressive medications with dental and periodontal treatment need in patients after solid organ transplantation. Transpl. Infect. Dis..

[6] Chapple I.L.C., Mealey B.L., Van Dyke T.E., Bartold P.M., Dommisch H., Eickholz P., Geisinger M.L., Genco R.J., Glogauer M., Goldstein M. (2018). Periodontal health and gingival diseases and conditions on an intact and a reduced periodontium: Consensus report of workgroup 1 of the 2017 World Workshop on the Classification of Periodontal and Peri-Implant Diseases and Conditions. J. Periodontol..

[7] Marshall R.I., Bartold P.M. (1999). A clinical review of drug-induced gingival overgrowths. Aust. Dent. J..

[8] Matsuda S., Okanobu A., Hatano S., Kajiya M., Sasaki S., Hamamoto Y., Iwata T., Ouhara K., Takeda K., Mizuno N. (2019). Relationship between periodontal inflammation and calcium channel blockers induced gingival overgrowth-a cross-sectional study in a Japanese population. Clin. Oral Investig..

[9] Rapone B., Ferrara E., Santacroce L., Cesarano F., Arazzi M., Liberato L.D., Scacco S., Grassi R., Grassi F.R., Gnoni A. (2019). Periodontal Microbiological Status Influences the Occurrence of Cyclosporine-A and Tacrolimus-Induced Gingival Overgrowth. Antibiotics.

[10] Hatahira H., Abe J., Hane Y., Matsui T., Sasaoka S., Motooka Y., Hasegawa S., Fukuda A., Naganuma M., Ohmori T. (2017). Drug-induced gingival hyperplasia: A retrospective study using spontaneous reporting system databases. J. Pharm. Health Care Sci..

[11] Paixão C.G., Sekiguchi R.T., Saraiva L., Pannuti C.M., Silva H.T., Medina-Pestana J., Romito G.A. (2011). Gingival overgrowth among patients medicated with cyclosporin A and tacrolimus undergoing renal transplantation: A prospective study. J. Periodontol..

[12] Ellis J.S., Seymour R.A., Taylor J.J., Thomason J.M. (2004). Prevalence of gingival overgrowth in transplant patients immunosuppressed with tacrolimus. J. Clin. Periodontol..

[13] Cota L.O., Aquino D.R., Franco G.C., Cortelli J.R., Cortelli S.C., Costa F.O. (2010). Gingival overgrowth in subjects under immunosuppressive regimens based on cyclosporine, tacrolimus, or sirolimus. J. Clin. Periodontol..

[14] Seymour R.A., Thomason J.M., Ellis J.S. (1996). The pathogenesis of drug-induced gingival overgrowth. J. Clin. Periodontol..

[15] Jung J.Y., Jeong Y.J., Jeong T.S., Chung H.J., Kim W.J. (2008). Inhibition of apoptotic signals in overgrowth of human gingival fibroblasts by cyclosporin A treatment. Arch. Oral Biol..

[16] Lauritano D., Moreo G., Limongelli L., Palmieri A., Carinci F. (2020). Drug-Induced Gingival Overgrowth: The Effect of Cyclosporin A and Mycophenolate Mophetil on Human Gingival Fibroblasts. Biomedicines.

[17] Todaro G.J., Green H. (1963). Quantitative studies of the growth of mouse embryo cells in culture and their development into established lines. J. Cell Biol..

[18] Beaumont J., Chesterman J., Kellett M., Durey K. (2017). Gingival overgrowth: Part 1: Aetiology and clinical diagnosis. Br. Dent. J..

[19] Hallmon W.W., Rossmann J.A. (1999). The role of drugs in the pathogenesis of gingival overgrowth. A collective review of current concepts. Periodontology 2000.

[20] Cotrim P., Martelli-Junior H., Graner E., Sauk J.J., Coletta R.D. (2003). Cyclosporin A induces proliferation in human gingival fibroblasts via induction of transforming growth factor-beta1. J. Periodontol..

[21] Ponnaiyan D., Jegadeesan V. (2015). Cyclosporine A: Novel concepts in its role in drug-induced gingival overgrowth. Dent. Res. J..

[22] Yamaguchi M., Naruishi K., Yamada-Naruishi H., Omori K., Nishimura F., Takashiba S. (2004). Long-term cyclosporin A exposure suppresses cathepsin-B and -L activity in gingival fibroblasts. J. Periodontal Res..

[23] Mariotti A., Hassell T., Jacobs D., Manning C.J., Hefti A.F. (1998). Cyclosporin A and hydroxycyclosporine (M-17) affect the secretory phenotype of human gingival fibroblasts. J. Oral Pathol. Med..

[24] Lauritano D., Palmieri A., Lucchese A., Di Stasio D., Moreo G., Carinci F. (2020). Role of Cyclosporine in Gingival Hyperplasia: An In Vitro Study on Gingival Fibroblasts. Int. J. Mol. Sci..

[25] Gagliano N., Moscheni C., Tartaglia G.M., Selleri S., Chiriva-Internati M., Cobos E., Torri C., Costa F., Pettinari L., Gioia M. (2008). A therapeutic dose of FK506 does not affect collagen turnover pathways in healthy human gingival fibroblasts. Transplant. Proc..

[26] Nassar C.A., Nassar P.O., Andia D.C., Guimarães M.R., Spolidorio L.C. (2008). The effects of up to 240 days of tacrolimus therapy on the gingival tissues of rats—A morphological evaluation. Oral Dis..

[27] Pamuk F., Cetinkaya B.O., Ayas B., Keles G.C., Gacar A. (2015). Evaluation of gingival alterations in rats medicated with cyclosporine A, tacrolimus and sirolimus: A stereological study. J. Periodontal. Res..

[28] Sam W.J., Chamberlain C.E., Lee S.J., Goldstein J.A., Hale D.A., Mannon R.B., Kirk A.D., Hon Y.Y. (2011). Associations of ABCB1 3435C>T and IL-10-1082G>A polymorphisms with long-term sirolimus dose requirements in renal transplant patients. Transplantation.

[29] Zhang X., Wang Z., Fan J., Liu G., Peng Z. (2011). Impact of interleukin-10 gene polymorphisms on tacrolimus dosing requirements in Chinese liver transplant patients during the early posttransplantation period. Eur. J. Clin. Pharmacol..

[30] Crews K.R., Hicks J.K., Pui C.H., Relling M.V., Evans W.E. (2012). Pharmacogenomics and individualized medicine: Translating science into practice. Clin. Pharmacol. Ther..

[31] García M., Macías R.M., Cubero J.J., Benítez J., Caravaca F., Gervasini G. (2013). ABCB1 polymorphisms are associated with cyclosporine-induced nephrotoxicity and gingival hyperplasia in renal transplant recipients. Eur. J. Clin. Pharmacol..

[32] Relling M.V., Klein T.E. (2011). CPIC: Clinical Pharmacogenetics Implementation Consortium of the Pharmacogenomics Research Network. Clin. Pharmacol. Ther..

